# Gd(III) and Yb(III) Complexes Derived from a New Water-Soluble Dioxopolyazacyclohexane Macrocycle

**DOI:** 10.1021/acsomega.3c03454

**Published:** 2023-09-11

**Authors:** Rosa E. Navarro, Alan Coronado, Motomichi Inoue, Ángel U. Orozco Valencia, Yedith Soberanes, Alex J. Salazar-Medina

**Affiliations:** † Departamento de Investigación en Polímeros y Materiales, Universidad de Sonora, Hermosillo 83000, Sonora, México; ‡ Centro de Investigación en Alimentación y Desarrollo, A. C., Hermosillo 83304, Sonora, México

## Abstract

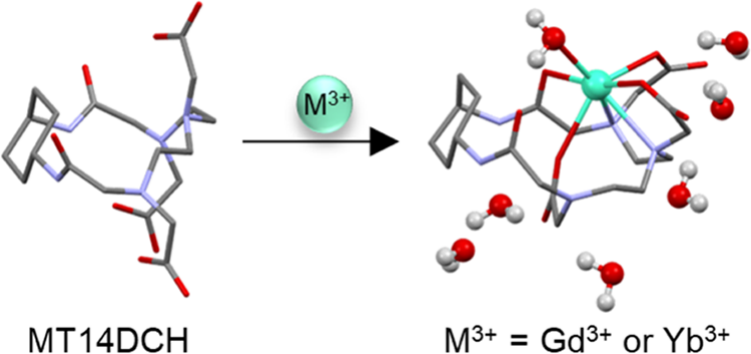

A new macrocyclic ligand was synthesized by a reaction between diethylenetriaminepentaacetic (DTPA) dianhydride and *trans*-1,4-diaminocyclohexane, and the Gd(III) and Yb(III) complexes were prepared. The compounds were characterized by spectroscopic methods. Structural calculation by DFT shows that the amide linkages are arranged in such a way that a conformational strain is minimized in the macrocyclic frame. The coordination modes of the ligand and water in the metal complexes were also determined by DFT. The longitudinal relaxation time *T*_1_ was measured for aqueous solutions of the Gd(III) complex. The *T*_1_ relaxivity arises from the structural feature that a water molecule coordinated to the paramagnetic metal is surrounded by a large open space, through which the exchange of water occurs readily to shorten the relaxation time of water in the entire region, as a result of the chelate conformation defined strictly by the amide groups and the cyclohexane ring.

## 1 Introduction

**Figure 1:**
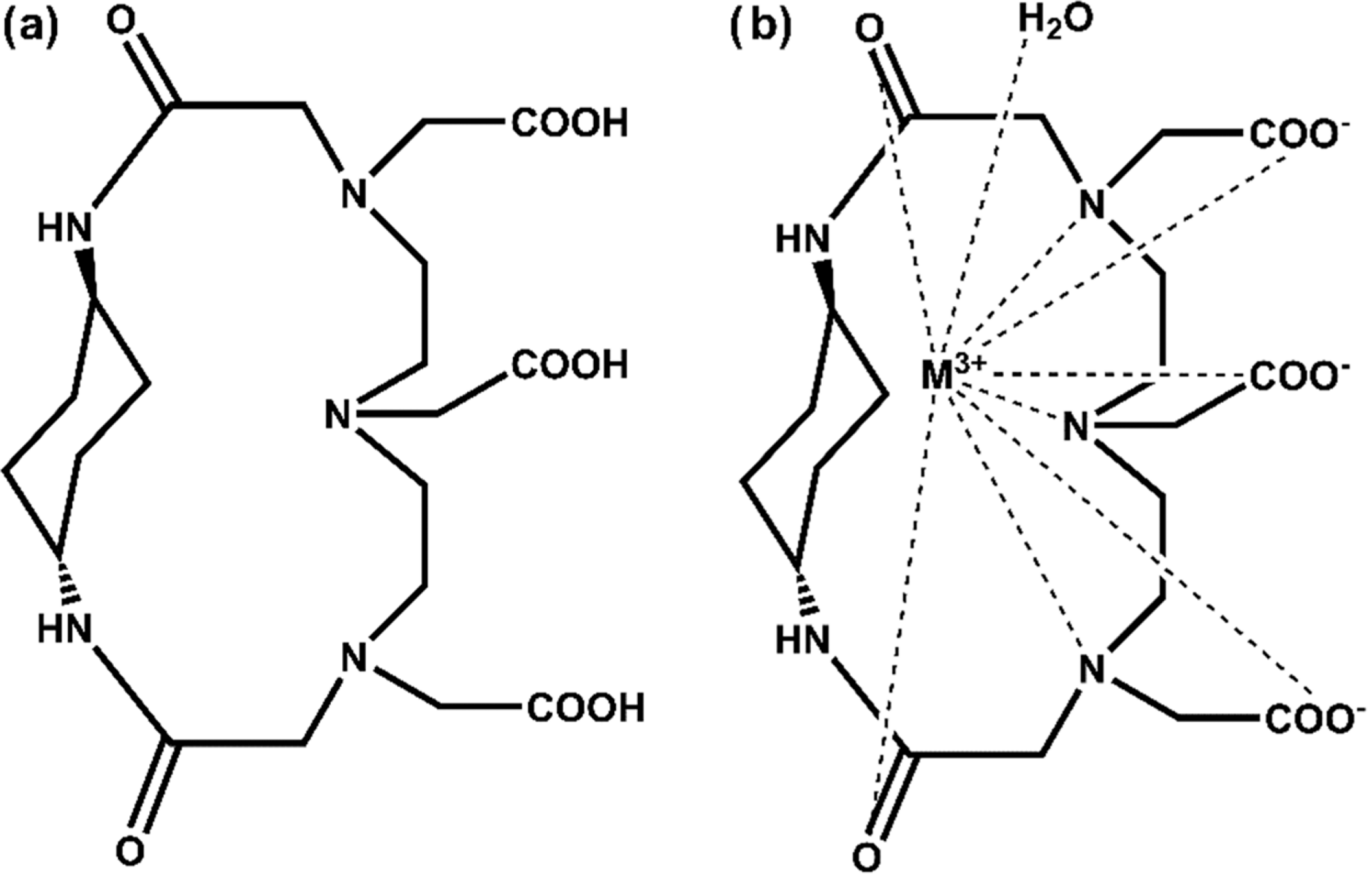
Structures proposed for (a) the ligand MT14DCH and (b) its metal complexes, M^3+^ = Gd^3+^ or Yb ^3+^. In the metal complexes, a water molecule is coordinated to the central metal ion at the axial position.

Paramagnetic lanthanide metal complexes are expected to be potentially applicable into a variety of clinical diagnoses.^1^ One of the most important practical examples is the Gd^3+^ complex of DTPA (diethylenetriaminepentaacetate) as a prominent contrast agent in magnetic resonance imaging (MRI).^2−4^ The serious toxicity of Gd^3+^ ions is suppressed by the coordination of the chelate, and the large paramagnetic moment effectively shortens the relaxation times of water protons in tested tissues to enhance the quality of images. Better contrast agents are still demanded.^3,5^ The desired agents are expected to be obtained from metal chelates of macrocycles rather than open-chain ligands because the formers generally have higher thermodynamic stability to yield a minor quantity of Gd^3+^ in the organism after administration.^6^ This strategy would be commonly useful for the design of metal-based diagnostic agents. As a part of such efforts, a variety of DTPA-derived macrocycles have been synthesized by cyclization between DTPA dianhydride and aromatic diamines.^7^ The geometry of the chelating rings is readily designed by the use of rigid aromatic diamines; the introduction of the aromatic group, however, decreases water solubility and may cause additional toxicity. To overcome this dilemma, the present study has employed an aliphatic diamine having a firmly defined conformation, *i.e*., *trans*-1,4-diaminocyclohexane. The new ligand is 1,13-(*trans*-cyclohexane-1,4)-2,12-dioxo-1,4,7,10,13-pentaaza-4,7,10-cyclotridecanetriacetic acid, abbreviated as MT14DCH (Figure 1). The Gd(III) and Yb(III) complexes have been characterized by spectroscopic methods, and the geometries have been optimized by DFT. Preliminary evaluation as an MRI agent in vitro has been made by determining the longitudinal relaxation time.

## 2 Results and Discussion

### 2.1 Synthesis and Characterization of MT14DCH and the Gd(III) and Yb(III) Complexes

The macrocyclic ligand MT14DCH has been synthesized by a reaction between DTPA dianhydride and *trans*-1,4-diaminocyclohexane in equimolar amounts (Figure S1). The reaction at high dilution under a nitrogen atmosphere promotes the cyclization and prevents polymerization, and the elevated reaction temperature improves the chemical yield. The ligand isolated as light-brown powder is highly soluble in water, in comparison to their aromatic analogue which requires a basic medium for entire solubilization.^7^ The Gd(III) and Yb(III) complexes are obtained in solution by a reaction of the ligand with appropriate metal carbonates, within a pH range from 6.0 to 6.4; they are abbreviated as Gd(MT14DCH) and Yb(MT14DCH), respectively.

The formation of the new compounds has been confirmed as detailed in Section 4; the primary characterization data are summarized in Table 1. The structure of the ligand is presented in Figure 1a; the equimolar cyclization of DTPA dianhydride and *trans*-1,4-diaminocyclohexane is corroborated by the maximum-abundant peak at the *m*/*z* of 470 in the ESI^–^ mass spectrum (Figure S2). Figure 1b shows the possible molecular structure of the metal complexes; the ligand molecule has eight potential coordination sites, *i.e.*, three carboxylate groups, three tertiary amines, and two amide carbonyls. The coordination of a water molecule is concluded from the mass spectra of the metal complexes (Figures S3 and S4). The structural features of the nonaromatic ligand endow the metal complexes with high water solubility at room temperature, which is desirable for the use as MRI contrast agents and other diagnostic agents.

**Table 1:** Characterization Data of MT14DCH and Its Gd(III) and Yb(III) Complexes

	MT14DCH·3H_2_O	Gd(MT14DCH)·9H_2_O	Yb(MT14DCH)·11H_2_O
state/color	solid/light brown	solid/light brown	solid/light brown
yield	50%	66%	70%
M. p.	113 °C	n/a	n/a
decomp. p.	222 °C	354 °C	356 °C
water solubility^a^	10	8	2
% found (C, H, N)	46.0, 7.4, 13.0	30.7, 5.7, 9.0	28.1, 5.5, 8.4
% calcd (C, H, N)	45.7, 7.5, 13.3	30.4, 6.1, 8.8	28.6, 6.2, 8.3
*m*/*z* (%)	470 (100)^b^	645 (100)^c^	659 (100)^d^

a g/100 g at 25 °C.

b [L – H]^−^.

c [ML·H_2_O + H]^+^ of ^158^Gd.

d [ML·H_2_O – H]^−^ of ^174^Yb.

The FTIR spectra of the ligand and the metal complexes are shown in Figure S5. The spectrum of the ligand exhibits an intense signal at 1706 cm^–1^ characteristic of the stretch of the carboxyl group. In addition, the N–H stretch band of the secondary amide is observed at 3258 cm^–1^, and amide I and II bands appear at 1622 and 1532 cm^–1^, respectively. These observations support the formation of the macrocycle with amide bonds. In the IR spectra of the Gd(III) and Yb(III) complexes, the N–H stretch band of the secondary amide is shifted to 3226 and 3242 cm^–1^, respectively, from the band of the ligand, and amide I and II bands as well as the stretch band of the carboxyl group are also shifted in the same manner, as a result of the coordination to the central metal in the complexes. The observation of broad bands in the range of 3250–3500 cm^–1^ suggests the presence of water molecules in the coordination sphere of each metal complex.

### 2.2 Thermogravimetric Analysis

The thermal decomposition patterns were observed by the thermogravimetric method with the objective of determining the modes of involved water molecules as well as calculating the metal contents in the complexes. The obtained thermograms, the decomposition steps, and the assigned fractions are shown in Figures S6–S8. The thermogram of the ligand (Figure S6) shows a change in mass between 25 and 182 °C corresponding to the weight of three water molecules. The following weight loss between 182 and 308 °C corresponds to the liberation of two pendant carboxyl arms. The DTPA fraction of the macrocyclic frame was released between 308 and 478 °C, resulting in the complete degradation between 478 and 630 °C.

The thermograms of the Gd(III) and Yb(III) complexes exhibit the first weight loss that corresponds to the liberation of six and seven water molecules, respectively (Figures S7 and S8). In the corresponding temperature range, the mass remains constant above 150 °C in either case, and hence, it is difficult to distinguish between water in the first coordination sphere and water of crystallization, although the former is confirmed by the mass spectra. Both metal complexes show similar thermal degradation patterns, in which the second mass change corresponds to the weight of the DTPA faction of the macrocyclic frame. The residual mass above 500 °C is attributable to the metal oxides: the residue of 24.0% for the Gd(III) complex is due to Gd_2_O_3_, giving the Gd content of 20.8% (calculated value, 21.4%); the Yb_2_O_3_ residue 27.4% is equivalent to 23.7% of Yb (calcd, 23.1%). These thermal data are consistent with the results of the CHN analyses.

### 2.3 NMR and Protonation Constants of the Ligand

**Figure 2:**
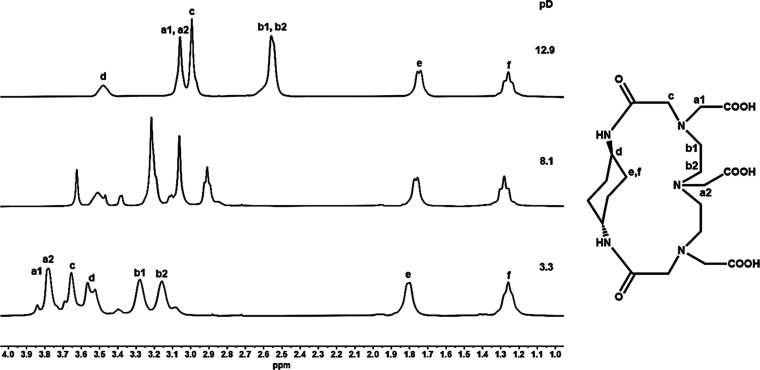
^1^H NMR spectra of MT14DCH at different pD values (*T* = 2 5 °C; 400 MHz; DSS). The signals are assigned as labeled in the molecular structure.

**Figure 3:**
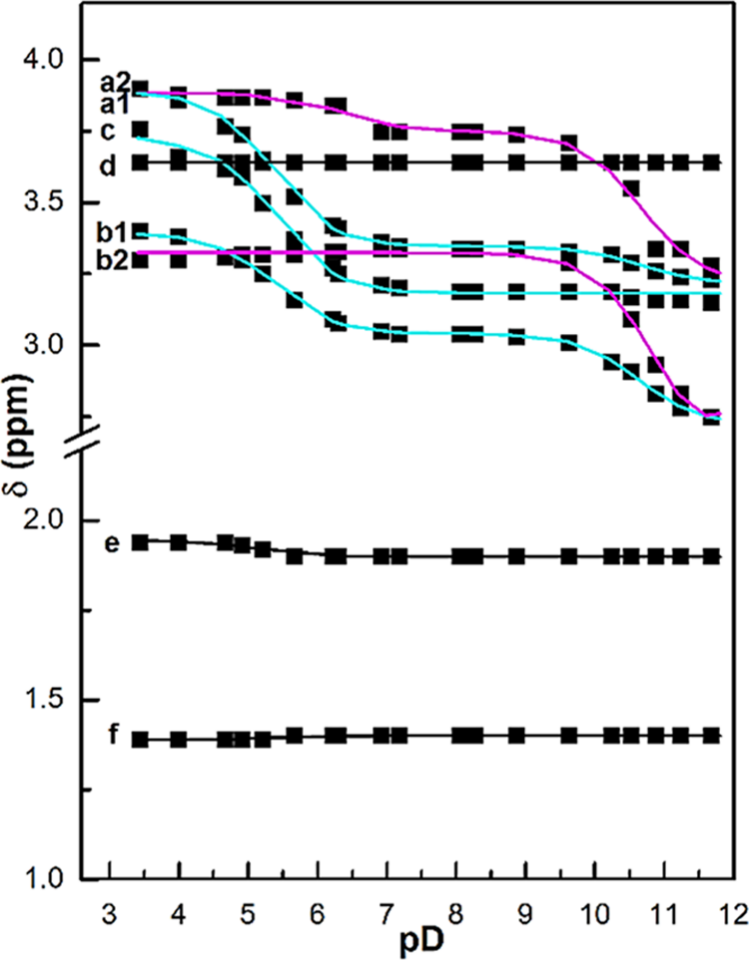
pD dependence of the ^1^H NMR shift δ observed for MT14DCH in D_2_O (the labels of protons are shown in Figure 2). The solid lines present the best fits of eq 1 with the protonation constants of log *K*_*D*1_ = 10.72 and log *K*_*D*2_ = 5.36. The shifts of protons a2 and b2 are sensitively changed upon the first protonation (the lines in magenta); protons a1 and c are sensitive to the second protonation step, and proton b1 responds to both steps (the lines in cyan).

Figure 2 presents the ^1^H NMR spectra of MT14DCH in D_2_O at selected pD values, and Figure 3 plots the chemical shifts δ of proton signals as a function of pD. Two peaks at δ 1.26 and 1.74 are due to cyclohexane ring protons at axial and equatorial positions, labeled f and e, respectively.^8^ Other signals were assigned with the aid of ^1^H–^1^H COSY and ^1^H–^13^C HSQC, which are shown in Figures S9 and S10, respectively. The δ values of protons in the DTPA moiety are sensitively changed with pD variation, responding to protonation at the donor sites, whereas the cyclohexane ring protons are little shifted with pD. These pD dependencies are consistent with the signal assignment based on the two-dimension spectra. The pD dependence caused by protonation at donor sites in the DTPA moiety is formulated as a function of pD by the following equations.^9^
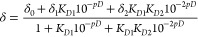
1Here, *K*_*D*1_ and *K*_*D*2_ are the first and second protonation constants in D_2_O, and δ_*j*_ (*j* = 0, 1, or 2) is the chemical shift intrinsic of the *j*-protonated species. By least-squares fitting, the protonation constants in D_2_O are determined as log *K*_*D*1_ = 10.72 based on the shifts of a2 and b2 protons, and log *K*_*D*2_ = 5.36 based on a1, b1, and c protons.

Protonation reduces electron density at the proton-accepting atom. As a result, the signal of the neighboring proton exhibits a downfield shift.^10^ The observed pD dependence, therefore, details the mode of protonation at each step. In the pD range of the first-protonation step, the largest change in δ is shown by a2 and b2 protons (lines in magenta) which are adjacent to the central amino nitrogen in the DTPA moiety, and the second largest change is found for b1 proton adjacent to the terminal amino nitrogen. In the pD range of the second protonation equilibrium, a1, b1, and c show large changes in δ (lines in cyan). These behaviors suggest that the first protonation occurs at the central amino nitrogen and the second protonation at the pair of the terminal nitrogen atoms. Upon the second protonation, the electron density at the central amino nitrogen is decreased due to the electrostatic repulsion between the added protons so that the b2 proton signal is shifted to the opposite field.^9,11,12^ The bond polarization produced by protonation attenuates in propagation along aliphatic linkages so rapidly that d, e, and f protons in the cyclohexane moiety exhibit little δ changes. A molecule carrying multiple protonation sites may form microspecies having different protonation status, and microequilibrium occurs between all possible combinations so that each site may have a fractional proton population.^13^ In order to obtain a clearer view of the protonation modes and identify species formed at each protonation step, the populations of acid hydrogen are calculated from the intrinsic δ_*j*_ values in eq 1, by assuming the following equation^13^
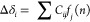
2Here, *f*_*j*_(*n*) is the proton population on the protonation site *j* in the *n*th protonation step and *C*_*ij*_ is the proportional constant. With the constants proposed for polyaminopolycarboxylates,^13^ proton populations were calculated on the terminal *N*-carboxymethyl group (Nt and Ot) and the central *N*-carboxymethyl group (Nc and Oc), as explained in Figure S11. For the first-protonation species LH^2–^, *f*_Nt_(1) = 0.1, *f*_Nc_(1) = 0.8, and *f*_Ot_(1) = *f*_Oc_ (1) = 0.0; for the species LH_2_^–^, *f*_Nt_(2) = 0.6, *f*_Nc_(2) = 0.7, and *f*_Ot_(2) = *f*_Oc_(2) = 0.0. The semiquantitative results suggest that the major species around pD 7 is protonated at the central amino nitrogen atom.

### 2.4 Thermodynamic Stability

The thermodynamic stability of Gd(III) and Yb(III) complexes was determined by performing a competition batch titration with DTPA as a competing ligand. The spectrophotometric titration experiments were performed to determine the conditional stability constants as the difference in pM values (M = Gd or Yb) between the competing DTPA and the ligand MT14DCH. The pM is a pH analogue and corresponds to the −log of the free metal ion concentration in the solution (eq 3), under specific standard conditions (pH 7.4, 25 °C, and 0.1 M KCl).
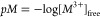
3The *pM* values, based on the experimental results, were calculated according to the following equilibria (eqs 4 and 5), where *L* is the ligand (MT14DCH), *C* is the competing ligand (DTPA), and M is the metal ion, Gd(III) or Yb(III).
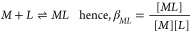
4
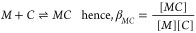
5The difference in log β_*ML*_ and β*_MC_* is equivalent to the difference in *pM* values (eq 6).
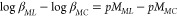
6
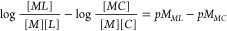
7
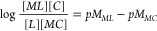
8
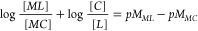
9Rearranging the previous equation gave eq 11, used to generate the log/log plots and determine Δ*pM* between the ligand and its competitor log ([*C*]/[*L*]) when log ([*MC*]/[*ML*]) = 0, that is, when the metal complexes generated between the ligand and competitor are generated in equal proportions.
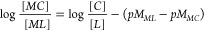
10
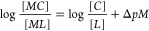
11The resulting concentrations of the free and complexed ligand after the competition process were obtained from the absorption spectra of the free ligand (MT14DCH), the metal complexes (GdMT14DCH and YbMT14DCH), and the mixtures in the presence of different amounts of DTPA (Figure S12a,b). From the plots of log ([*M*-DTPA]/[*M*-*L*]) *vs* log ([DTPA]/[*L*]) (Figure S12c,d), the differences in *pM* between MT14DCH and DTPA (log ([DTPA]/[*L*])) when ([*M*-DTPA]/[*M*-*L*]) = 0 were calculated. The *p*Gd and *p*Yb values of DTPA are known to be 19.1 and 19.4, respectively.^14−16^ The *p*Gd value of the MT14DCH ligand was calculated to be 16.7, and the *p*Yb value was 16.0. These values are lower than that reported for DTPA but slightly higher than DTPA–BMA (*p*Gd = 15.8), a clinically used contrast agent.^14^ Although the cyclization process reduced the *pM* values of the DTPA, the MT14DCH ligand maintains competitive conditional stability constants when Gd(III) and Yb(III) complexes were synthesized.

### 2.5 Theoretical Calculation

**Figure 4:**
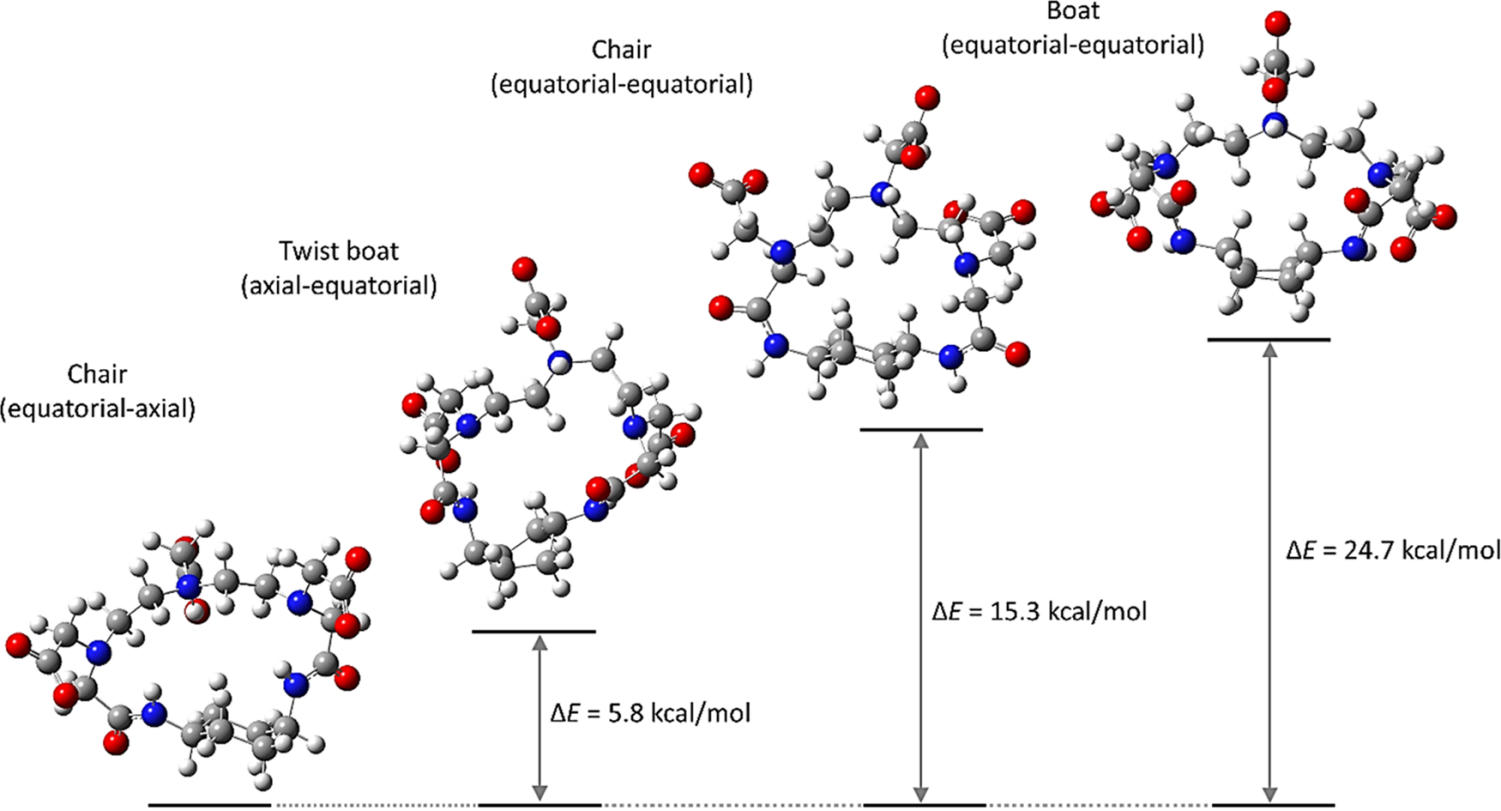
Optimized structures of the ligand MT14DCH with different conformations of the cyclohexane moiety.

The geometry optimization was performed by the DFT method for the ligand in the LH^2–^ state in which the central amino nitrogen is protonated, as the *p*D-variation NMR has shown that it is the major species in a wide pH range including the physiological pH. Four optimized structures having different conformations of the cyclohexane ring were obtained as presented in Figure 4. In the most stable structure, the amide linkages of cyclohexane with DTPA are formed by one nitrogen atom at the axial position and the other at the equatorial position to minimize possible strain in the macrocyclic framework. The conformation in which both amide nitrogen atoms are equatorial causes the tension of the macrocyclic framework, increasing the molecular energy. The conformations of the boat form also have high molecular energies compared with the corresponding chair conformations.

**Figure 5:**
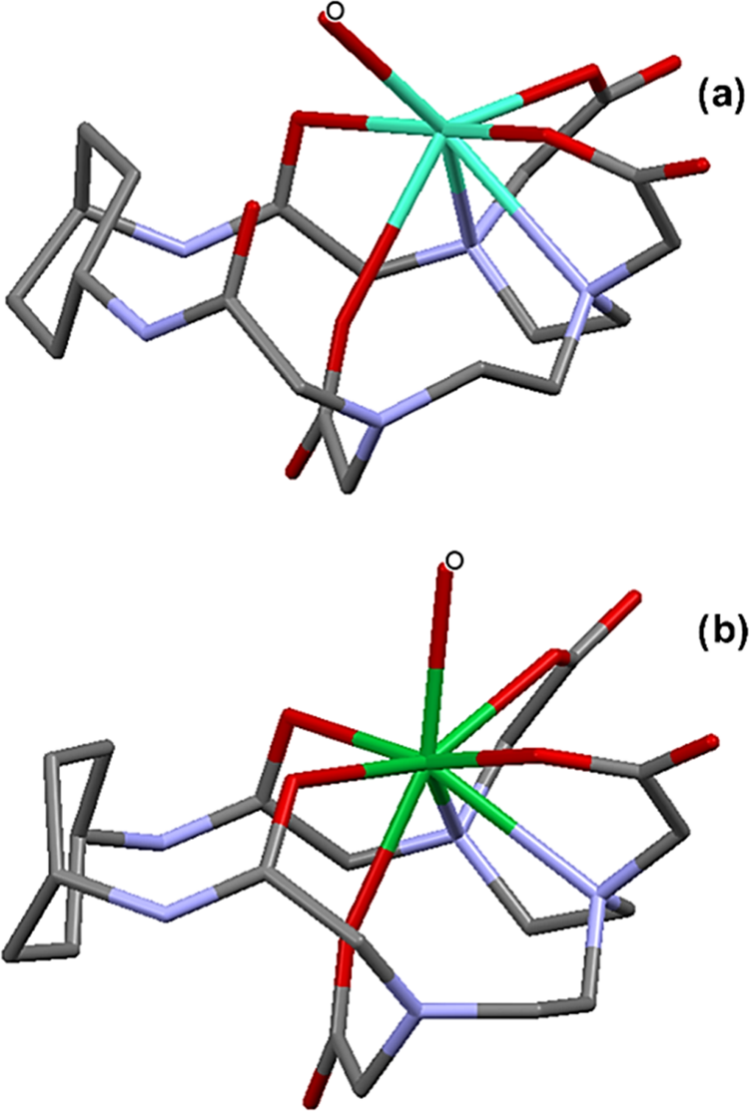
Structures optimized for the complexes by the DFT method: (a) Gd(MT14DCH)·H_2_O and (b) Yb(MT14DCH)·H_2_O. The atom labeled O is the oxygen atom of a water molecule axially coordinated. Hydrogen atoms from macrocycles are not shown for clarity.

Figure 5 shows the optimized structures of the solvated complexes Gd(MT14DCH)·H_2_O and Yb(MT14DCH)·H_2_O; selected bond lengths and bond angles are collected in Table 2. In Gd(MT14DCH)·H_2_O (Figure 5a), the central metal forms seven coordinate bonds, and six of them originate from the macrocycle, *i.e*., one amide carbonyl oxygen atom, two amino nitrogen atoms, and three acetate oxygen atoms. The seventh coordination site is occupied by water oxygen. Two carboxylate groups, from the central arm and one of the terminal arms, are coordinated to the central metal on the same plane, whereas the other terminal group forms a coordinate bond at the opposite side.

**Table 2:** Bond Lengths (Å) and Bond Angles (°) of the Gd(III) and Yb(III) Complexes of MT14DCH^a^

bond type	Gd(MT14DCH)·H_2_O	Yb(MT14DCH)·H_2_O
**M–O(CO)**	2.50, 2.57, 2.79	2.35, 2.35, 2.41
**M–O(CN)**	2.67, 3.87	2.42, 2.48
**M– O(H**_**2**_**O)**	2.76	2.57
**M–N**	2.80, 2.81, 4.42	2.66, 2.70, 3.67
**∠O(CO)–M–O(CO)**	97, 103, 127	91, 110, 136
**∠O(CO)–M–O(CN)**	59, 72, 73, 95	73, 77, 82, 89,
**∠O(CO)–M–O(H**_**2**_**O)**	74, 102, 130	66, 68, 155
**∠O(CO)–M–N**	34, 63, 64	67, 66, 44
**∠N–M–O(CN)**	45, 60	62, 60
**∠N–M– O(H**_**2**_**O)**	89, 132, 153	120, 123, 130
**∠N–M–N**	55, 68, 90	61, 71, 104

a M, central metal; −O(CO), carboxylate oxygen; −O(CN), amide oxygen; and −O(H_2_O), water oxygen.

This mode of coordination has been reported for the X-ray structures of the Gd(III) complexes of analogous macrocyclic ligands.^17−19^ In Yb(MT14DCH)·11H_2_O, eight coordinate bonds are formed (Figure 5b). The coordination mode is basically the same as in the Gd(III) complex, but two amide groups participate in the coordination to construct seven coordinate bonds in total. The eighth site is occupied by a water molecule.

### 2.6 Longitudinal Relaxation Time (*T*_1_)

The relaxation time of water molecules involved in a tissue is shortened by the addition of paramagnetic ions so that the contrast of magnetic resonance images is improved.^3−5^ The potential capacity as a contrast agent is described by the relaxivity, which is determined from the concentration dependence of the relaxation times. The inverse of the longitudinal relaxation time *T*_1_ observed for the solvent is expressed by the sum of the diamagnetic term intrinsic of the solvent and the term due to the paramagnetic ion effect, as follows.
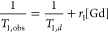
12Here, *T*_1,*d*_ is the relaxation time of the solvent in the absence of the paramagnetic agent and the effect of the paramagnetic solute (gadolinium complex in this case) is presented by the relaxivity *r*_1_ in the second term. The *T*_1_ values were measured for solutions of Gd(MT14DCH) in water at 80 MHz and in deuterium oxide at 400 MHz with changing the concentration of the complex. The obtained *T*_1_ values collected in D_2_O are shown in Table S3. The relaxivity was determined to be 5.02 mm^–1^s^–1^ (mm = mmol kg^–1^) from the linear plot based on eq 12 (Figure S13). For comparison with the reported values, it is converted to 5.56 mM^–1^s^–1^ (mM = mmol dm^–3^) with the density of the solvent.^20^ The *T*_1_ values observed in H_2_O are collected in Table S4, and the relaxivity is determined to be 4.59 mM^–1^s^–1^ from the linear plot (Figure S14). These relaxivities are of the same order of the magnitude as the value 4.84 mM^–1^s^–1^ in D_2_O and 3.38 mM^–1^s^–1^ in H_2_O calculated for Magnevist, which is a widely used commercial contrast agent derived from DTPA, when the frequency dependence of *r*_1_ is considered.^3^ The paramagnetic effect of a metal complex is perceived by a water molecule bonded to the metal ion and propagates over the entire system tested, through an exchange of water molecules. As found in Figure 5a, Gd(MT14DCH) involves a large open space around the coordinated water molecule. This structural feature facilities the water-exchange process and results in a competent relaxivity value.

## 3 Conclusions

A new macrocyclic ligand, MT14DCH, was synthesized by a reaction between DTPA dianhydride and *trans*-1,4-diaminocyclohexane in a yield of 50%. The formation of the macrocycle was confirmed by NMR, IR, and mass spectra as well as elemental analysis. The structure was determined by DFT calculation.

The ligand forms stable Gd(III) and Yb(III) complexes, in which a water molecule is included in the first coordination sphere. The coordination modes determined by DFT optimization show the formation of large space around the coordinated water molecule. This structural feature facilitates molecular exchange of water to result in the high *T*_1_ relaxivity.

## 4 Materials and Methods

### 4.1 Synthesis of the Ligand MT14DCH

The macrocyclic ligand MT14DCH was synthesized in a three-neck round-bottom flask assembled with a dropping funnel, a condenser, and a nitrogen-gas inlet tube, as follows. In the flask, 977 mg (2.73 mmol) of DTPA dianhydride was suspended in 60 mL of DMF and heated at a reaction temperature of 80 °C with constant magnetic stirring under a nitrogen atmosphere. Into the suspension, a 20 mL DMF solution containing 267 mg (2.33 mmol) of *trans*-1,4-diaminocyclohexane was added dropwise every 20 s through the dropping funnel in the period of approximately 2 h. The reaction system was kept for 24 h more. The solid formed was collected with a filter paper and washed with deionized water. An aqueous solution of the isolated solid was concentrated with a rotary evaporator at 70 °C under reduced pressure. The resulting amber viscous liquid was concentrated further on a hot plate, treated with acetone before dryness and left to stand for 24 h. The obtained whitish precipitate was separated by decantation and dried at 50 °C under reduced pressure. The dried precipitate was dissolved in hot water at 70 °C, and the resulting solution was filtered with paper, concentrated to one-fourth of the initial volume on a hot plate, and left to stand overnight until a precipitate was formed. The mother liquors were removed by decantation, and the solid product was dried at 50 °C under reduced pressure. A light brown powder was obtained in a yield of 50%. Melting point, 113 °C; decomposition point, 222 °C. ^1^H NMR (400 MHz, D_2_O, pD = 11.7, DSS): δ = 1.39 (m, 4H, H_f_), 1.90 (m, 4H, H_e_), 2.73 (s, 8H, H_b1_ and H_b2_), 3.16 (s, 4H, H_c_), 3.20 (s, 2H, H_a2_), 3.25 (s, 4H, H_a1_), and 3.63 (s, 2H, H_d_). ^13^C NMR (100 MHz, D_2_O, pD = 3.2): δ = 30.07 (C_e,f_), 48.09 (C_d_), 50.69 (C _b2_), 51.82 (C_b1_), 54.21 (C_c_), 56.70 (C_a1_), 56.93 (C_a2_), 166.86 (C_amide_), 170.28 (C_a1_–CO_2_^–^), 172.87 (C_a2_–CO_2_^–^). MS(ESI^–^) *m*/*z* (%): 470.02 (100) [M – H]^−^ for ^12^C and 471.01 (21)[M – H]^−^ for ^13^C. IR (ATR) ν/cm^–1^ = 3258 (vNH_amide_), 1706 (vCO_2_H), 1622 (amide I), 1532 (amide II). Elemental analysis, calcd (%) for C_20_H_33_N_5_O_8_·3H_2_O: C, 45.7; H, 7.5; N, 13.3. Found (%): C, 46.0; H, 7.4; N, 13.0.

### 4.2 Gd(III) and Yb(III) Complexes

For the synthesis of the Gd complex, 236 mg (0.50 mmol) of the ligand was dissolved in 10 mL of water (pH 3.0) and was mixed with 130 mg (0.29 mmol) of gadolinium carbonate Gd_2_(CO_3_)_3_. The mixture of pH 6.4 was heated at 50 °C with vigorous stirring for the first 4 h to promote the reaction and then at 40 °C for 20 h. The unreacted exceeding carbonate was removed by paper filtration, and the product was recrystallized from the aqueous solution by the addition of acetone after concentration, separated by decantation, and dried at 50 °C under reduced pressure. Yield: 66%. Decomposition point, 354 °C. MS(CI^+^) *m*/*z*: 645.15 (100) [M·H_2_O + H]^+^ of ^158^Gd; isotope satellites at 642.15 (^155^Gd), 643.15 (^156^Gd), 644.15 (^157^Gd), and 647.15 (^160^Gd). IR (ATR) ν/cm^–1^ = 3226 (vNH_amide_), 1574 (vCO_2_H, amides I and II). Relaxivity, *r*_1_/mM^–1^s^–1^: 4.66 in H_2_O at 80 MHz and 5.57 in D_2_O at 400 MHz. Elemental analysis, calcd (%) for GdC_20_H_30_N_5_O_8_·9H_2_O: C, 30.4; H, 6.1; N, 8.8. Found (%): C, 30.7; H, 5.7; N, 9.0.

The Yb(III) complex Yb(MT14DCH) was synthesized by the same method as for the Gd(III) complex, using 139 mg (0.50 mmol) of ytterbium carbonate Yb_2_(CO_3_)_3_. The pH of the resulting solution was 6.0. Yield, 70%. Decomposition point, 356 °C. MS(ESI^–^) *m*/*z*: 659.20 [M·H_2_O – H]^−^ for ^174^Yb; isotope satellites, 656.20 (^171^Yb), 657.18 (^172^Yb), 658.20 (^173^Yb), and 661.18 (^176^Yb). IR (ATR) ν/cm^–1^ = 3242 (vNH_amide_), 1574 (vCO_2_H, amides I and II). Elemental analysis, calcd (%) for YbC_20_H_30_N_5_O_8_·11H_2_O: C, 28.6; H, 6.2; N, 8.3. Found (%): C, 28.1; H, 5.5; N, 8.4.

### 4.3 Characterization Methods

The mass spectrum was obtained with an Agilent Technologies model 6130 Quadrupole LC/MS spectrometer by the electrospray ionization method in the negative mode. The high-resolution mass spectrum was obtained with a Bruker model SolariX 2xR mass spectrometer at the University of Arizona (Tucson, AZ).

The ^1^H and ^13^C NMR spectra were obtained for D_2_O solutions by the use of a Bruker Avance 400 NMR spectrometer operating at 400 MHz for ^1^H and 100 MHz for ^13^C at a probe temperature of 25 °C. The internal reference was sodium 3-trimethylsilyl-1-propanesulfonate (DSS).

The CHN analyses of the ligand and the complexes were carried out at ALS analysis (Tucson, AZ).

The infrared spectra of the solid samples were recorded on a PerkinElmer Frontier FTIR spectrometer equipped with an attenuated total reflection (ATR) adapter in the range of 4000–400 cm^–1^.

Thermogravimetric analysis was performed using a thermal balance, PerkinElmer model Pyris 1, to determine the water and metal contents: the heating rate was 10 °C/min for the ligand and 5 °C/min for the metal complexes; the temperature range was from 25 to 800 °C; and air flow was 20 mL/min.

### 4.4 Determination of Protonation Constants

The protonation sites as well as the protonation constants of the ligand were determined by ^1^H NMR titration in D_2_O. A 25 mM stock solution was prepared with D_2_O containing 0.1% DSS and divided to two parts. One was acidic showing *p*D 3.45. The second part was made basic with KOD/D_2_O up to a *p*D of 11.69. By mixing the two solutions in different ratios, a series of sample solutions of desired pD values were prepared in such a way that the ligand concentration was kept constant, and the ^1^H NMR spectra were recorded in turn. The least-squares fitting of a δ *vsp*D plot determined the protonation constants. The *p*D values were obtained by conversion of the pH values measured with a glass electrode on the basis of the relation *p*D = pH_meas_ + 0.45.^21^

### 4.5 Thermodynamic Stability

The thermodynamic stability of the MT14DCH lanthanide complexes was analyzed based on the procedure reported by Xu et al.,^14^ with slight modifications. Solutions of the Gd(III) and Yb(III) MT14DCH complexes were mixed with standardized DTPA solutions. The concentration of the complexes was kept constant at 3 × 10^–5^ M, and the concentration of the competing ligand was gradually increased in a range from 1:0.1 to 1:1 (L/DTPA). All of the solutions were prepared at identical volumes with HEPES buffer (pH = 7.4) and 0.1 M KCl, at 25 °C. The solutions were left with magnetic stirring for 24 h to guarantee a complete thermodynamic equilibrium. After incubation, the samples were analyzed by UV–vis spectroscopy on a PerkinElmer Lambda 20 Spectrophotometer. The concentrations of the complexed and free ligand were determined, in each solution, from the absorption spectra. Spectra of free and fully complexed ligands, under the same conditions, were used as references. The concentration values calculated were used the log/log plots to determine the difference in *pM* (*M* = Gd or Yb) between the competing DTPA and the ligand MT14DCH.

### 4.6 Longitudinal Relaxation Time (*T*_1_)

The longitudinal relaxation time *T*_1_ was determined for water molecules in aqueous solution of Gd(MT14DCH), by the use of Bruker Avance 400 and Magritek Spinsolve 80 NMR spectrometers at 400 MHz and 80 MHz, respectively. A stock solution of 7.76 × 10^–4^ molal of the complex was prepared with D_2_O. By diluting this solution, a series of sample solutions of different concentrations were prepared in a weight of 0.5 g. For measurements at 80 MHz, aqueous solutions were prepared in the same manner with ultrapure (Milli-Q) water. The employed program was T1IR with the 180°−τ–90° pulse sequence.

### 4.7 Computational Details

The theoretical structure of MT14DCH was modeled by geometry optimization with the atomic orbital base 6-311+G(2d,p) and the functional of exchange–correlation energy PBE0, using an implicit solvation with water through the polarizable continuum model (PCM). The theoretical structures of the Gd(III) and Yb(III) complexes were modeled with density functional theory (DFT).^22^ For the calculations, the functional of exchange–correlation energy PBE0^23^ was employed along with the atomic orbital base 6-31+G(d,p), which was included only for the light atoms. For the electronic treatment of the Gd(III) and Yb(III) ions were used the pseudopotential of the Stuttgart/Cologne family.^24^ In the case of gadolinium was used a *quasi*-relativistic effective core potential ECP for 54 internal electrons, ECP54MWB, together with an optimized Gaussian-type valence orbital base (7s6p5d)/[5s4p3d]-GTO.^25,26^ In the case of ytterbium was used a totally relativistic effective core potential for 68 internal electrons, ECP68MDF, along with a valence orbital base optimized and adjusted for relativistic correlations PP(2, MCDHF+Breit+QED).^27^ The molecular geometries of the Gd(III) and Yb(III) complexes were optimized by including an implicit solvation with water through the PCM.^28^ In addition, for a well representation of solvent medium, we also took into account nine and eleven explicit water molecules for Gd(III) and Yb(III) complexes, respectively. Every molecular optimization was verified to correspond to the energy minimum on the potential energy surface through a vibrational frequency analysis. All of the calculations were performed with the computational package Gaussian 09.^29^

## Supporting Information Available

Reaction scheme of the synthesis of the MT14DCH ligand; mass spectrometry, IR and 2D NMR spectroscopy; thermal decomposition analysis; acid proton population and determination of protonation constants; longitudinal relaxation time (*T*_1_); and computational details (DOCX)
