# Supplementary material for: Gd(III) and Yb(III) Complexes Derived from a New Water-Soluble Dioxopolyazacyclohexane Macrocycle
Source: ACS Omega. 2023 Sep 11;8(38):34575–82. doi: 10.1021/acsomega.3c03454 (PMC10536832; doi:10.1021/acsomega.3c03454)
Supplement: Supplementary file 1 — ao3c03454_si_001.docx [file ao3c03454_si_001.docx]

Supporting Information

**Gd(III) and Yb(III) complexes derived from a new water-soluble dioxopolyazacyclohexane macrocycle**

Rosa E. Navarro^1,2^, Alan Coronado^1^, Motomichi Inoue^1^, Ángel U. Orozco Valencia^1^, Yedith Soberanes^1^, and Alex J. Salazar-Medina^1^*

1. Departamento de Investigación en Polímeros y Materiales, Universidad de Sonora, Hermosillo Sonora 83000, México.

2. Centro de Investigación en Alimentación y Desarrollo, A. C. Hermosillo Sonora, 83304, México.

***** Corresponding Author: [alex.salazar@unison.mx](mailto:alex.salazar@unison.mx) (A.J.S.-M.); [yedith.soberanes@unison.mx](mailto:yedith.soberanes@unison.mx) (Y.S.);

Tel.: +52-662-259-2161.

**Synthesis of the MT14DCH ligand**


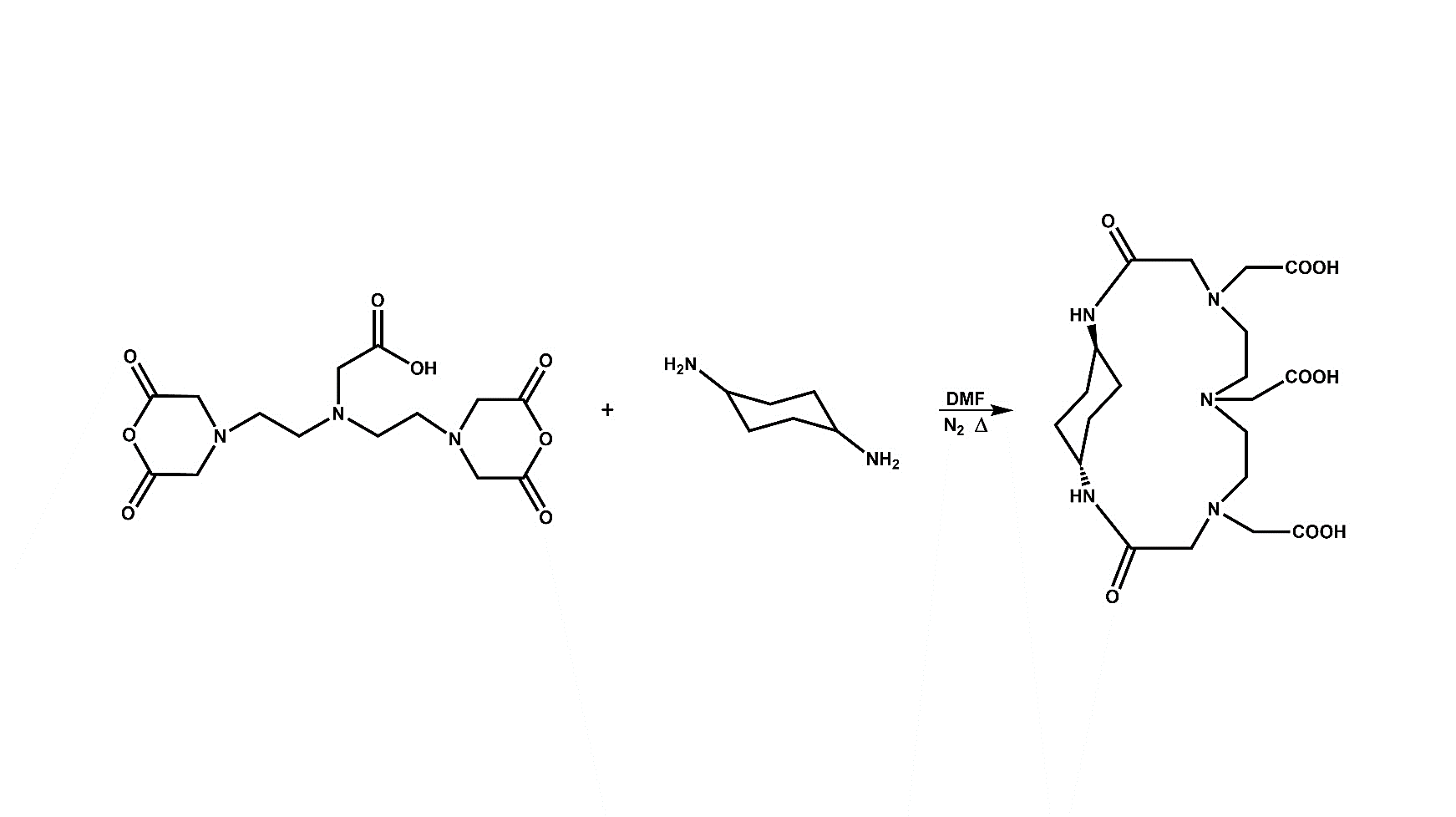


**Figure S1.** Reaction scheme between DTPA dianhydride and trans-1,4-diaminocyclohexane in the synthesis of the macrocyclic ligand MT14DCH.

**Mass spectra**


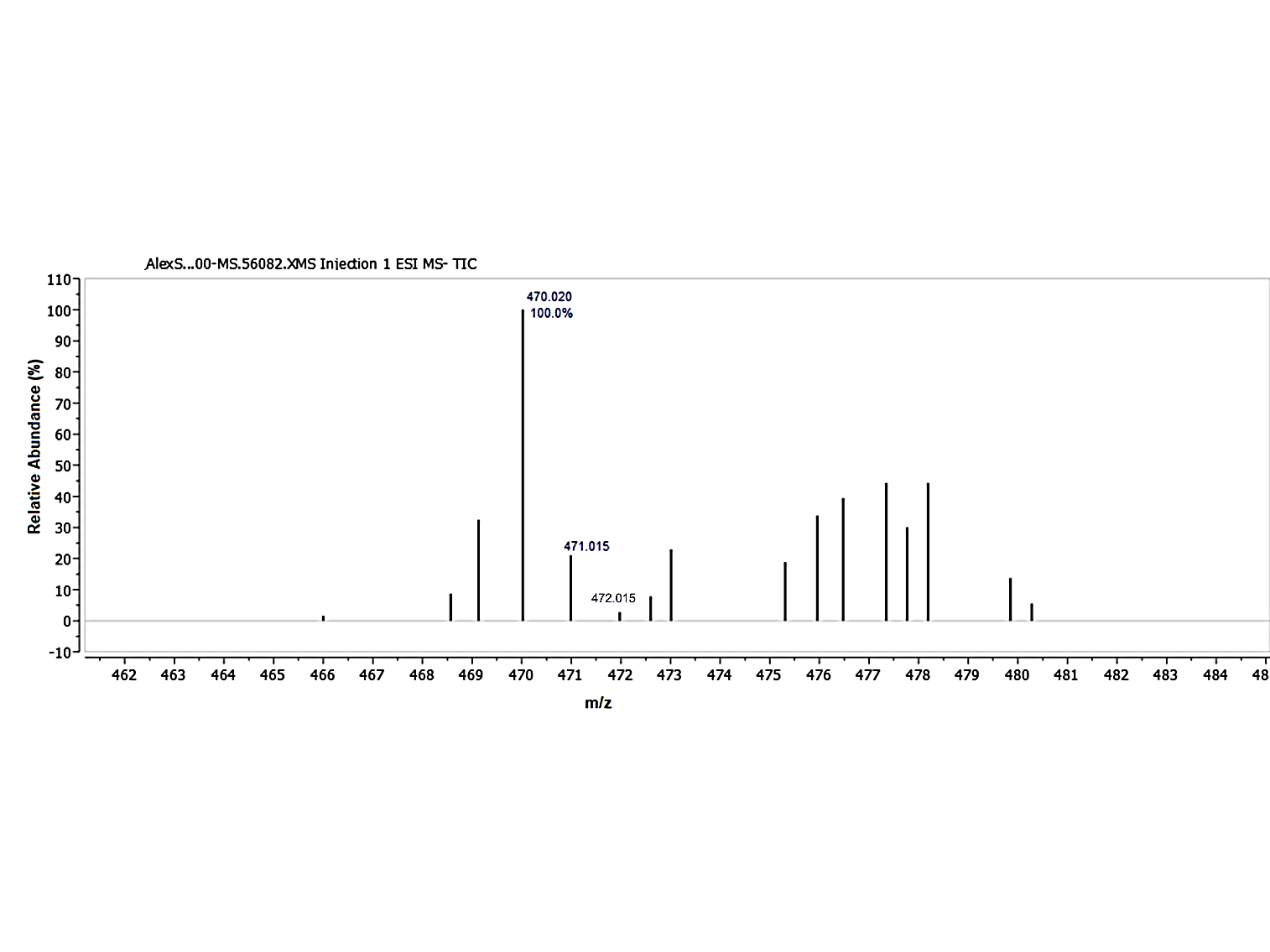


**Figure S2.** Mass spectrum of the ligand MT14DCH in an ESI¯ ionization mode; the 470 peak is assignable to [M − H]^−^ (calc. *m*/*z* = 470.22), accompanied by isotope peaks at 471 and 472.

**
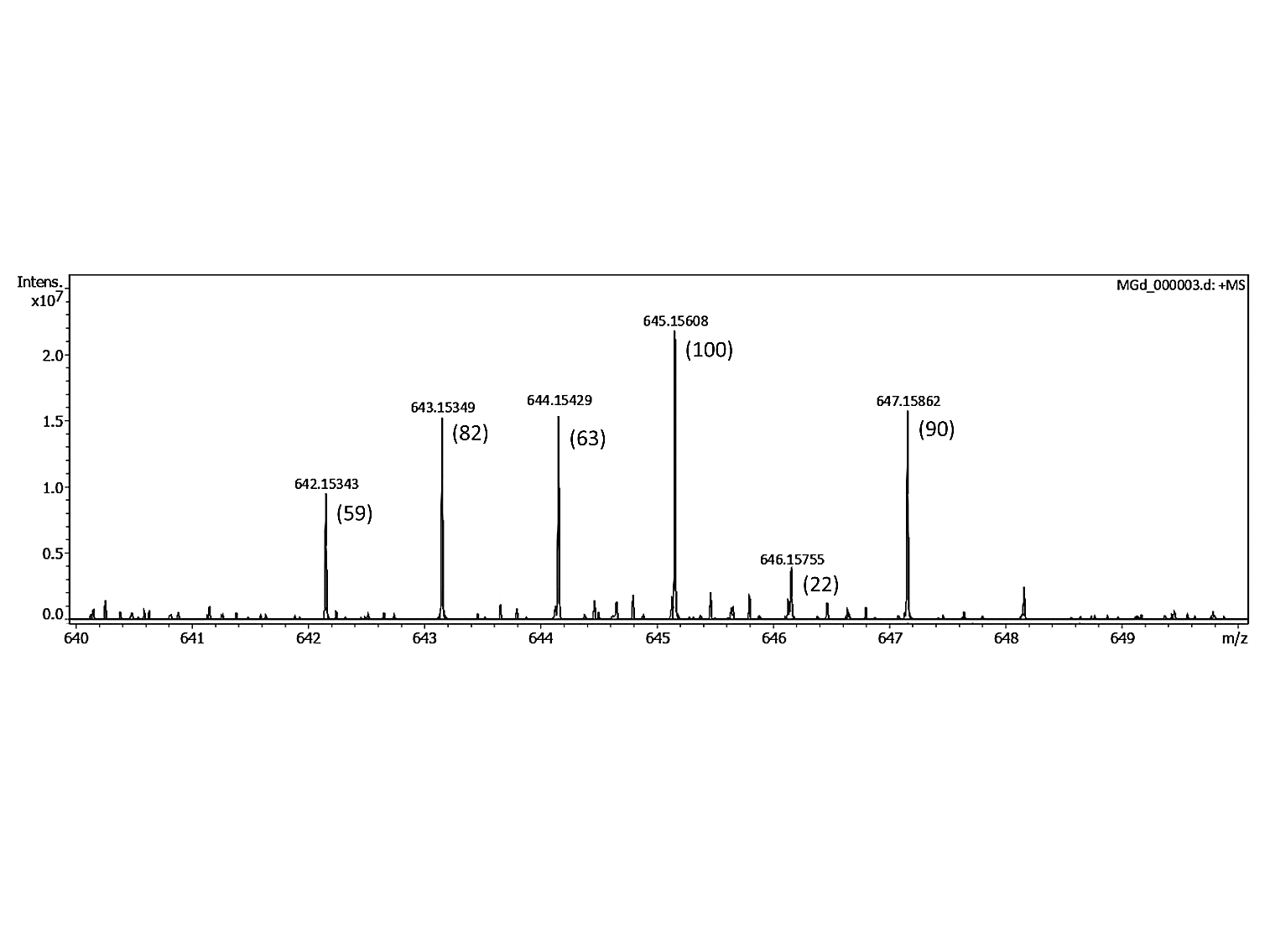
**

**Figure S3.** High resolution mass spectrum of the Gd complex obtained by positive chemical ionization. The peak at 645.15 is assigned to [^158^Gd(MT14DCH – 3H)·H_2_O + H]⁺ (Calc. 645.15). The numbers in the parentheses show the relative abundances predicted from the isotopes of Gd and C.

**
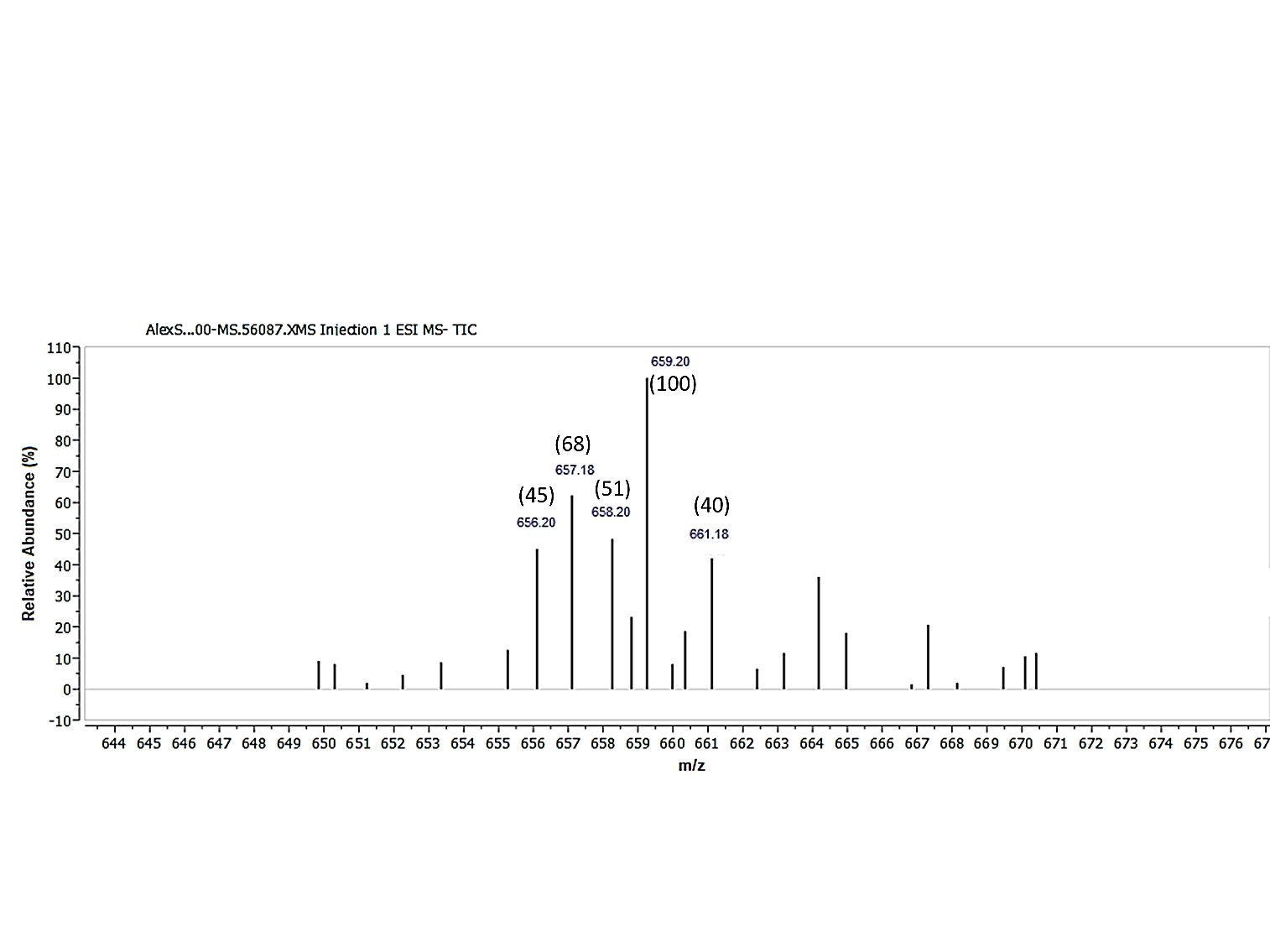
**

**Figure S4.** Mass spectrum of the Yb complex in an ESI¯ mode. The peak at 659.20 is assignable to [^174^Yb(MT14DCH – 3H)·H_2_O – H]^–^ (Calc. 659.15). The numbers in the parentheses show the relative abundances predicted from the isotopes of Yb and C.

**IR spectra**

**

**

**Figure S5.** FTIR spectra of ligand MT14DCH and the Gd(III) and Yb(III) complexes.

**Thermogravimetric analysis**


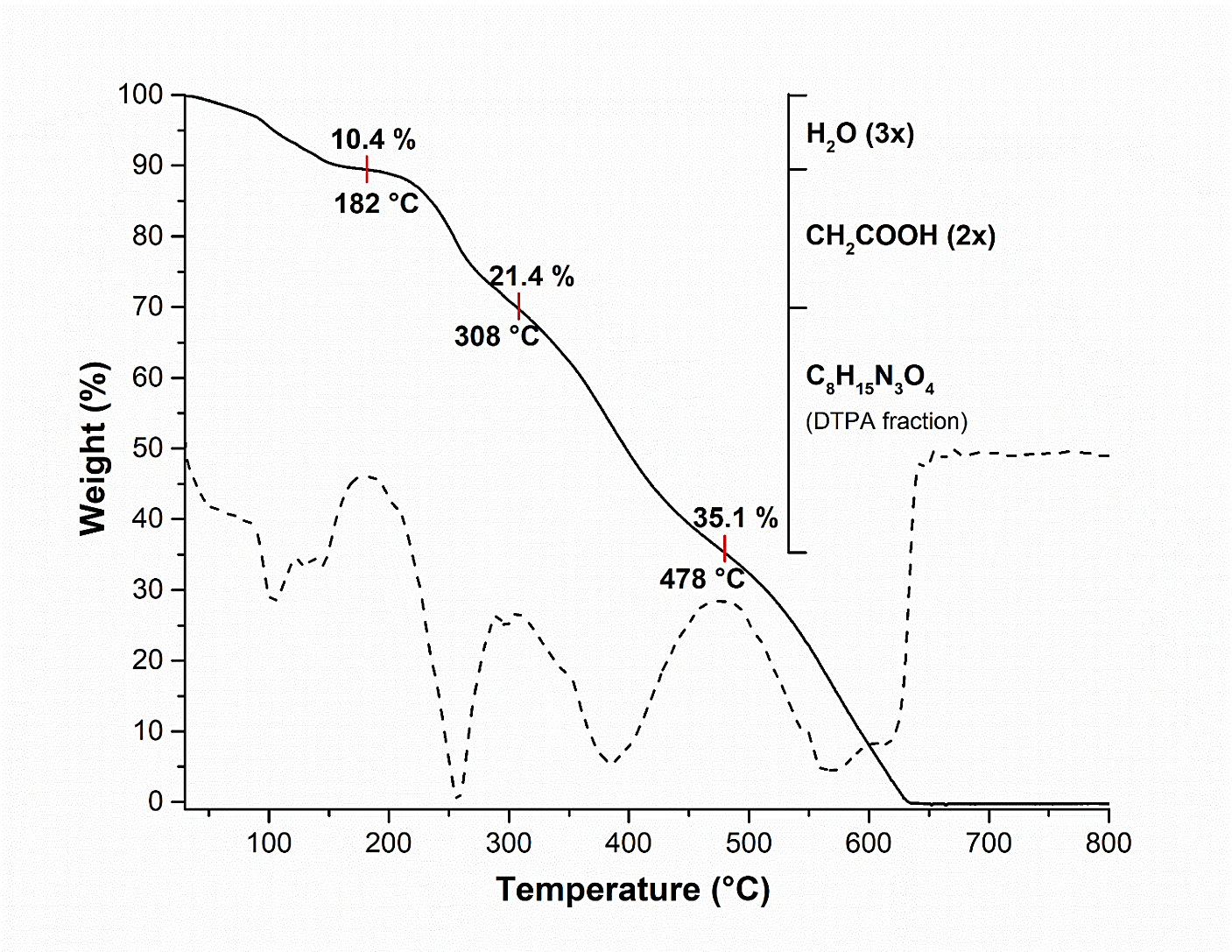


**Figure S6.** Thermal decomposition analysis of the ligand MT14DH. Marks in solid lines present the weight loss percentage and the temperature at each step of decomposition. Dashed line represents the derivative of the curve of weight loss (%/min).





**Figure S7.** Thermal decomposition analysis of the Gd(MT14DCH) complex. Marks in solid lines present the weight loss percentage and the temperature at each step of decomposition. Dashed line represents the derivative of weight loss (%/min).





**Figure S8.** Thermal decomposition analysis of the Yb(MT14DCH) complex. Marks in solid lines present the weight loss percentage and the temperature at each step of decomposition. Dashed line represents the derivative of weight loss (%/min).

**2D NMR analysis**

**
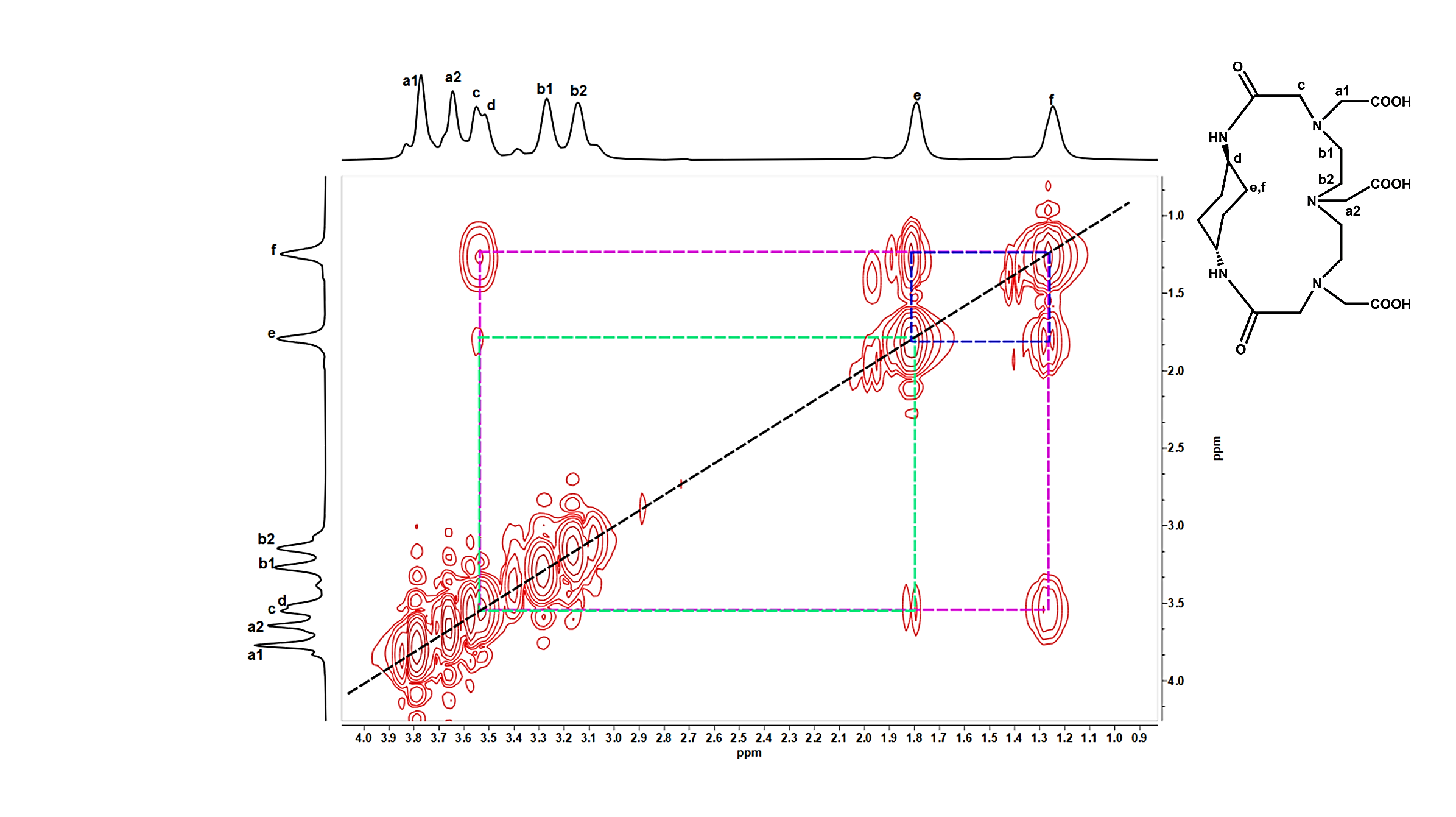
**

**Figure S9.** ^1^H-^1^H COSY NMR spectrum of MT14DCH ligand (400 MHz, D_2_O, pD 3.3). Correlation between axial and equatorial protons e and f in cyclophane are indicated by the blue dash-line square. Correlation between d proton and cyclohexane protons (e, f) are depicted by green and magenta dash-line squares.

**
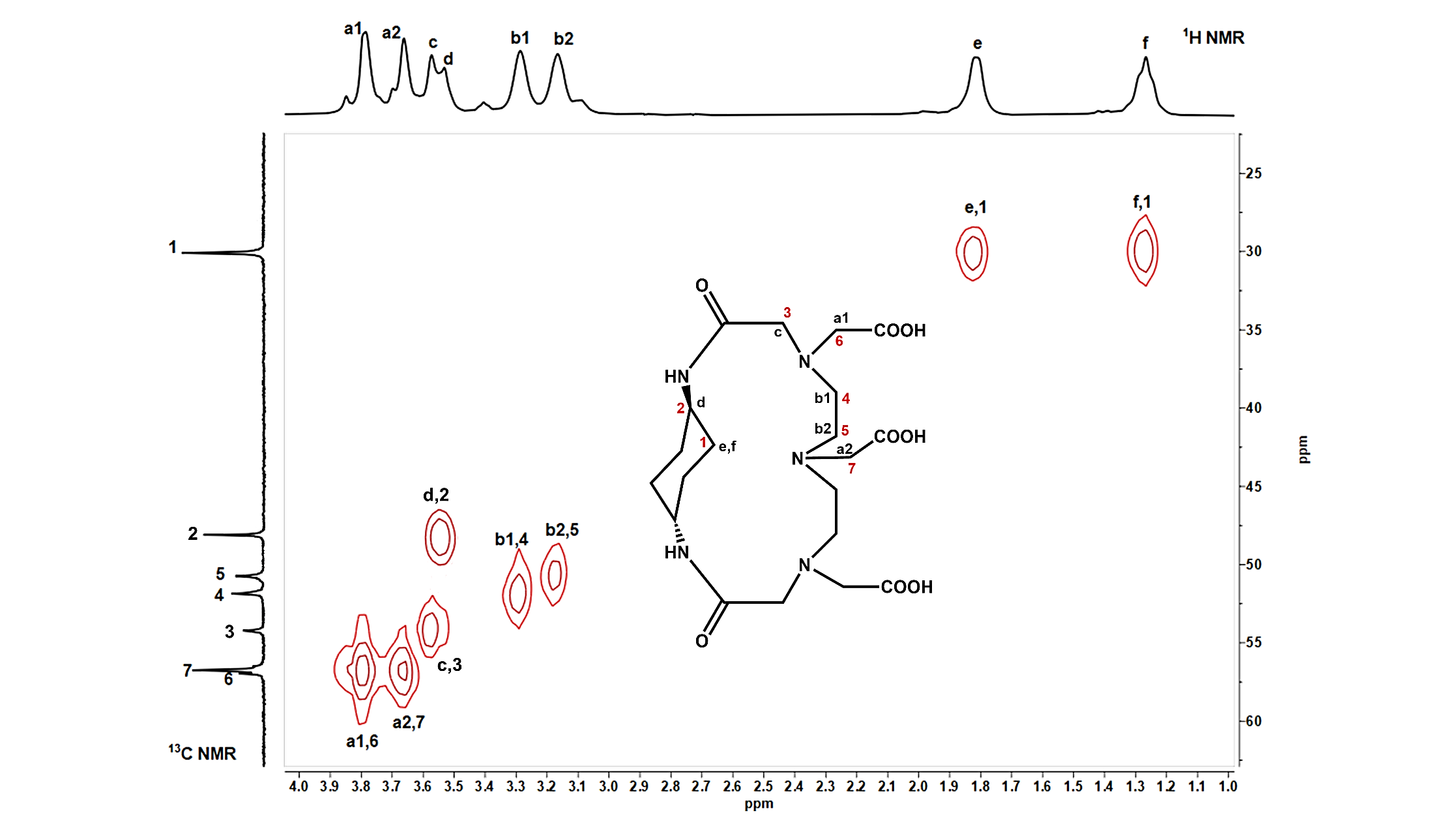
**

**Figure S10.** ^1^H-^13^C heteronuclear correlation (HSQC) spectrum of MT14DCH ligand (400 MHz, D_2_O, pD 3.2).

**Population of acid hydrogen**

The populations of acid hydrogen *f*_X_(*n*) at the donor site X in LH*_n_* were calculated by Equation 2 in the main text; the intrinsic *δ_i_* values in the equation were determined by curve fitting of observed *δ* versus pD plots with Equation 1. The calculation was made under the restriction *f*_Nc_(*n*) + *f*_Oc_(*f*_Nt_(*n*) + 2*f*_Nt_(*n*) + 2*f*_Ot_(*n*) = *n.* The scheme of population is shown below for each protonated species.


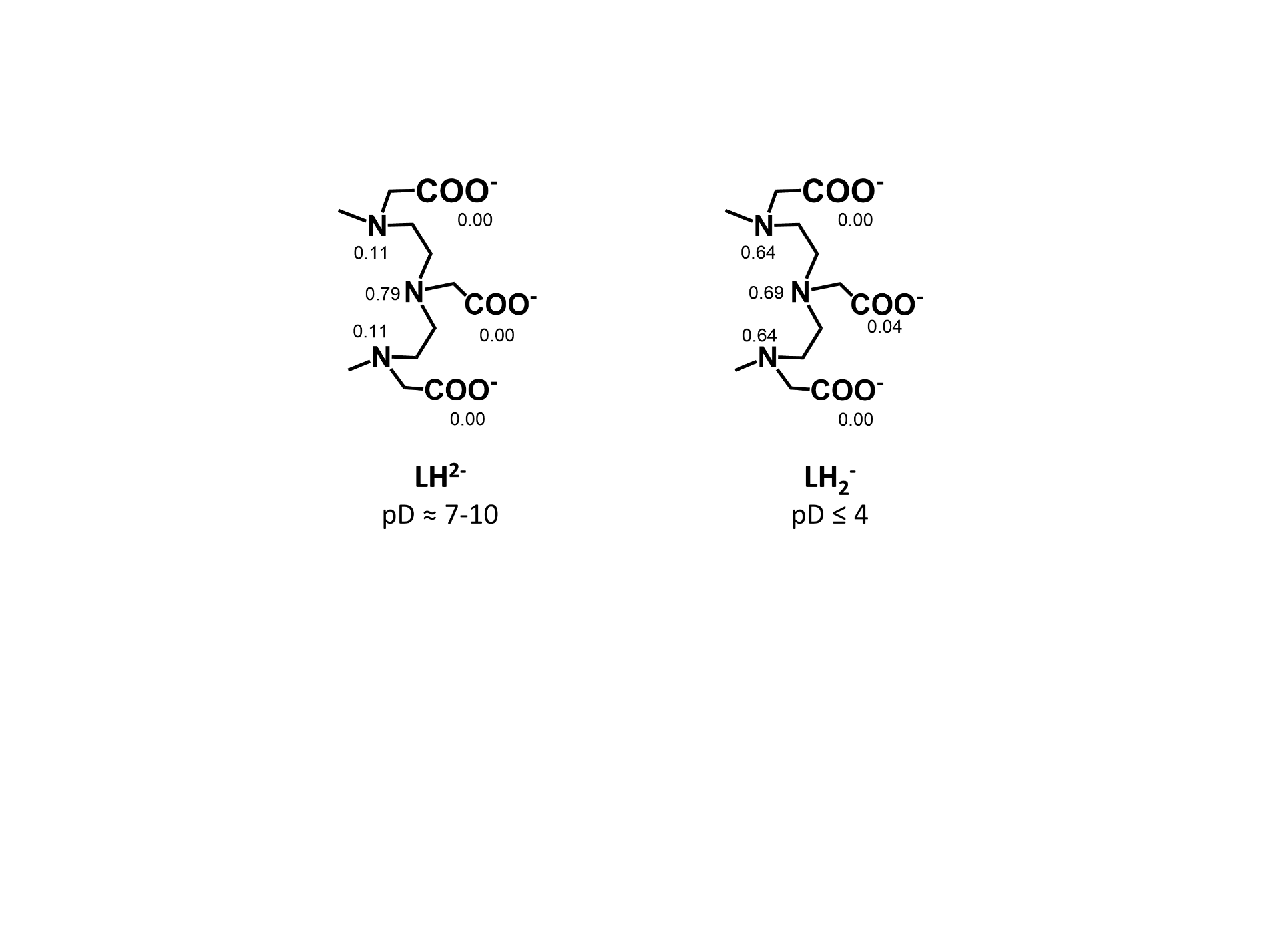


**Figure S11.** Schematic representation of acid proton population and microspecies in MT14DCH ligand.

**Thermodynamic stability**

**
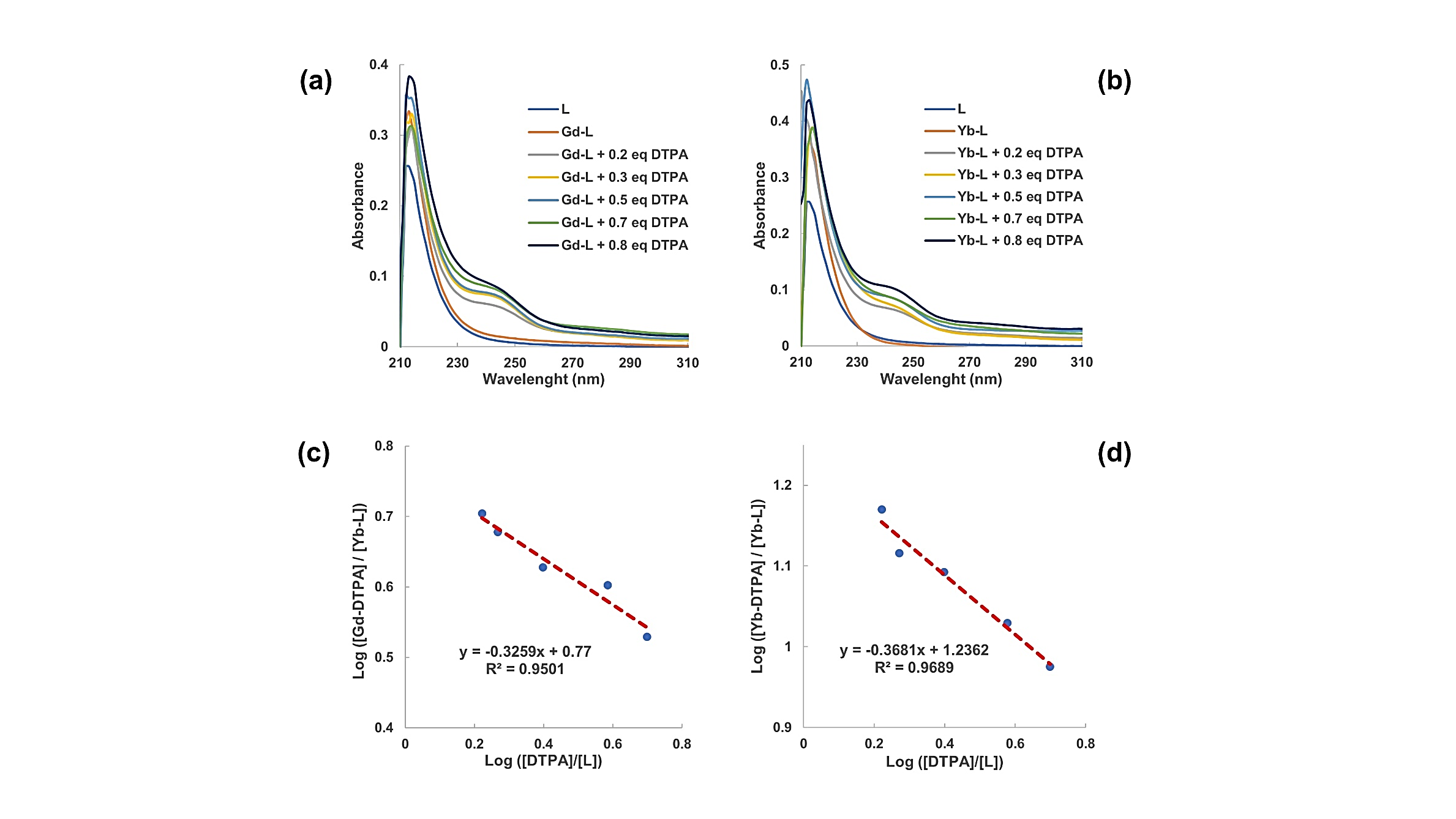
**

**Figure S12.** (a) and (b) are the absorption spectra of the competition titration of MT14DCH (L) *vs.* DTPA for Gd(III) and Yb(III), respectively. The concentration of the ligand and metal was kept constant at 3 x 10^-5^ M, at pH 7.4, 25 °C, and 0.1 M KCl. (c) and (d) correspond to the competition titration log/log plots of the ligand MT14DCH *vs.* DTPA; the difference in pM between MT14DCH and DTPA is indicated by the x-intercept.

**Relaxivity**


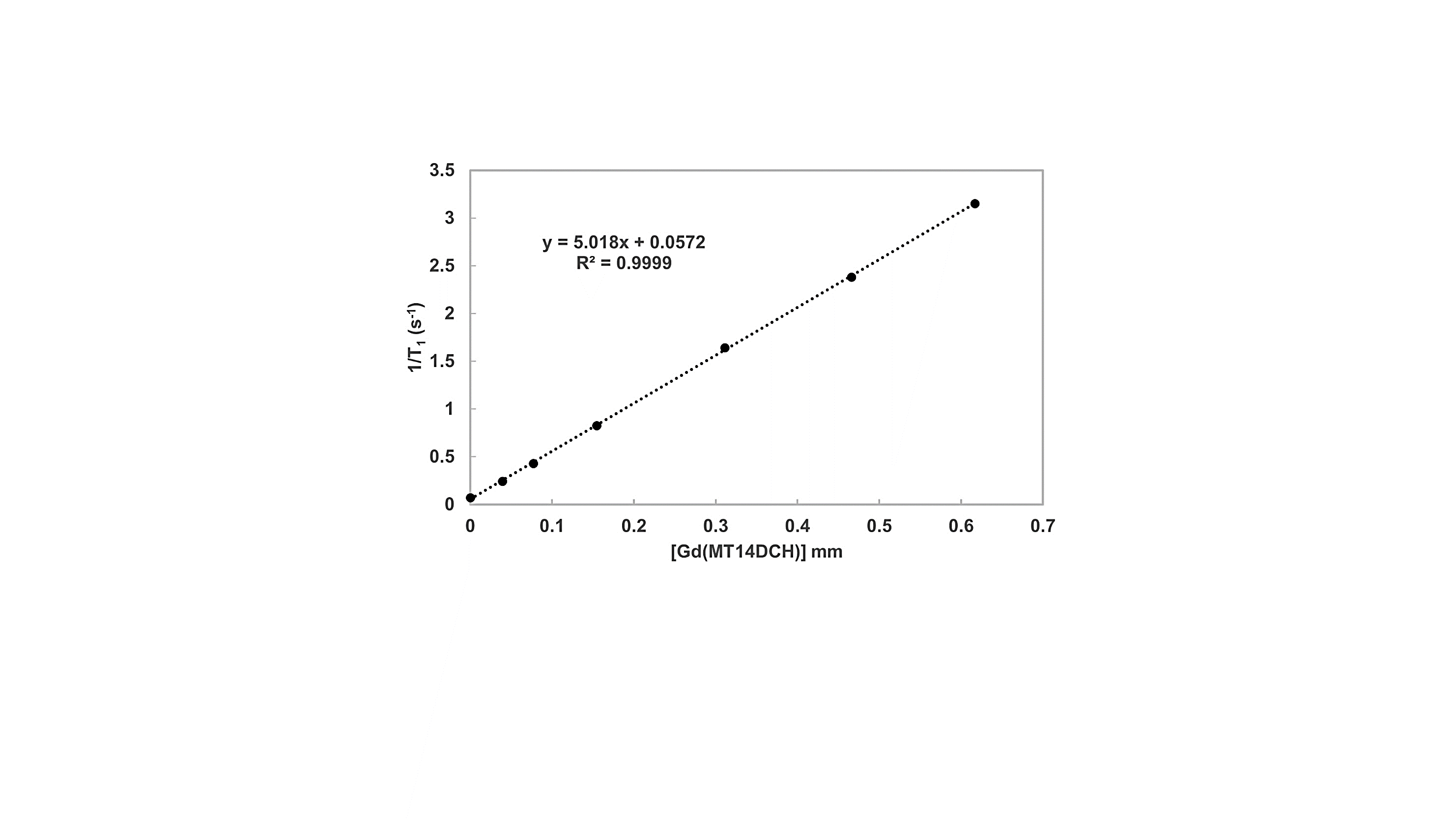


**Figure S13.** Inverse *T_1_* values (solid circles) of H_2_O at 400 MHz and 25 °C in D_2_O solutions of Gd(MT14DCH) complex at different concentrations. The dotted line represents a linear fit, the slope of which gives the longitudinal relaxivity 5.56 mM^−1^s^−1^.


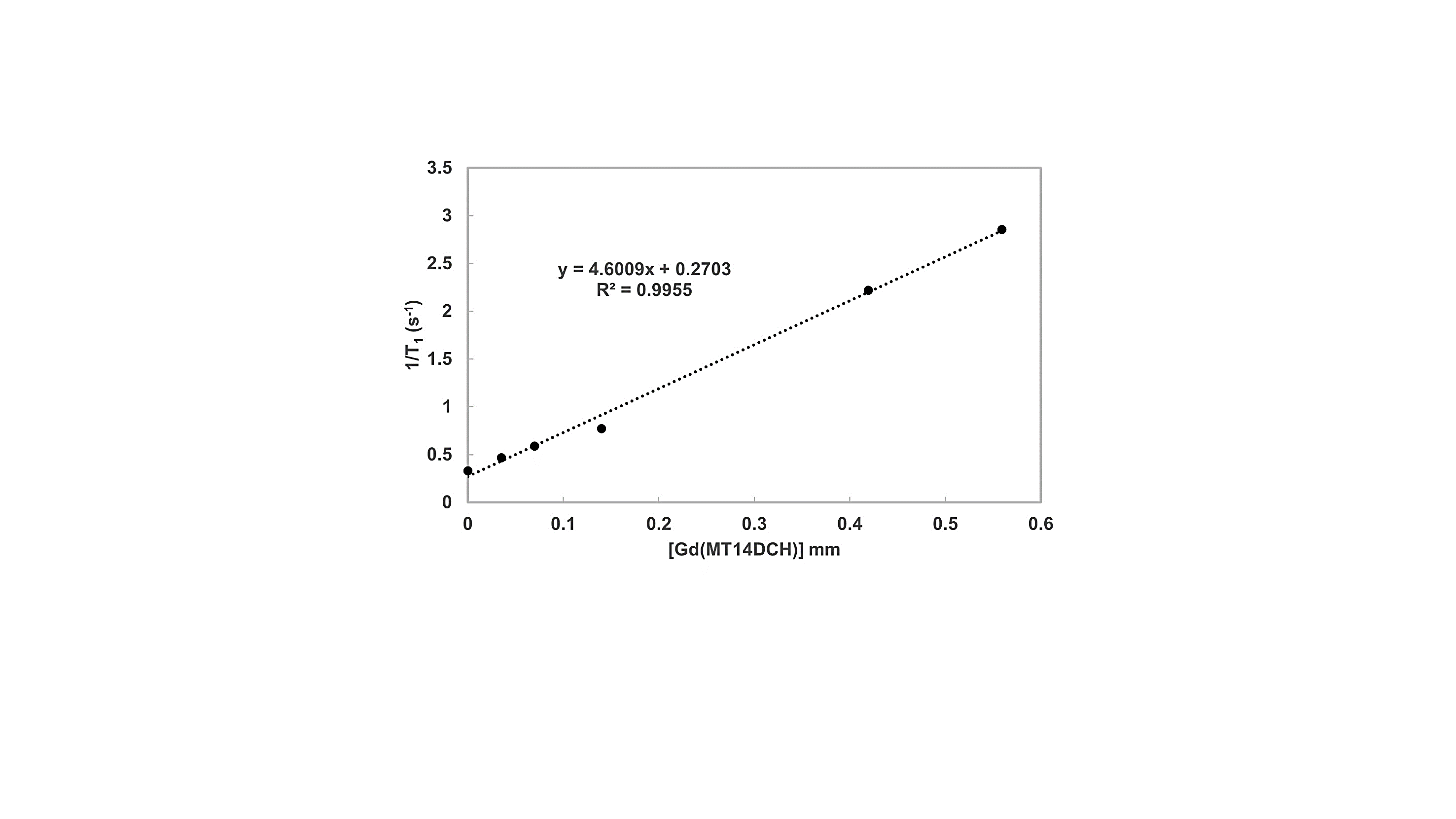


**Figure S14.** Inverse *T_1_* values (solid circles) of H_2_O at 80 MHz and 25 °C in Gd(MT14DCH) solutions at different concentrations. The dotted line represents a linear regression, the slope of which shows the longitudinal relaxivity as 4.59 mM^−1^s^−1^.

| **Table S1.** Proton assignment of the ^1^H NMR spectrum of the ligand MT14DCH at pD = 11.7 (*T* = 25 °C; 400 MHz; DSS). | | |
| --- | --- | --- |
| **δ (ppm)** | **Label** | **Integrated intensity** |
| 1.39 | **f** | 4 |
| 1.90 | **e** | 4 |
| 2.73 | **b1, b2** | 8 |
| 3.16 | **c** | 4 |
| 3.20 | **a2** | 2 |
| 3.25 | **a1** | 4 |
| 3.63 | **d** | 2 |

| **Table S2.** Chemical shifts (δ) of the protons of MT14DCH at different pD values in D_2_O solution. | | | | | | | | |
| --- | --- | --- | --- | --- | --- | --- | --- | --- |
| **pD** | **a2** | **a1** | **c** | **d** | **b1** | **b2** | **e** | **f** |
| 3.45 | 3.90 | 3.90 | 3.76 | 3.64 | 3.40 | 3.28 | 1.94 | 1.39 |
| 4.00 | 3.88 | 3.86 | 3.66 | 3.64 | 3.38 | 3.28 | 1.94 | 1.39 |
| 4.67 | 3.87 | 3.77 | 3.62 | 3.64 | 3.31 | 3.31 | 1.94 | 1.39 |
| 4.92 | 3.87 | 3.74 | 3.59 | 3.64 | 3.30 | 3.32 | 1.93 | 1.39 |
| 5.21 | 3.87 | 3.65 | 3.50 | 3.64 | 3.25 | 3.32 | 1.92 | 1.39 |
| 5.67 | 3.86 | 3.52 | 3.37 | 3.64 | 3.16 | 3.32 | 1.90 | 1.40 |
| 6.22 | 3.84 | 3.42 | 3.27 | 3.64 | 3.09 | 3.33 | 1.90 | 1.40 |
| 6.30 | 3.84 | 3.41 | 3.25 | 3.64 | 3.08 | 3.33 | 1.90 | 1.40 |
| 6.93 | 3.75 | 3.36 | 3.21 | 3.64 | 3.05 | 3.34 | 1.90 | 1.40 |
| 7.18 | 3.75 | 3.35 | 3.20 | 3.64 | 3.04 | 3.34 | 1.90 | 1.40 |
| 8.05 | 3.75 | 3.34 | 3.19 | 3.64 | 3.04 | 3.34 | 1.90 | 1.40 |
| 8.28 | 3.75 | 3.34 | 3.19 | 3.64 | 3.04 | 3.34 | 1.90 | 1.40 |
| 8.88 | 3.74 | 3.34 | 3.19 | 3.64 | 3.03 | 3.34 | 1.90 | 1.40 |
| 9.63 | 3.71 | 3.33 | 3.19 | 3.64 | 3.01 | 3.29 | 1.90 | 1.40 |
| 10.24 | 3.64 | 3.32 | 3.19 | 3.64 | 2.94 | 3.19 | 1.90 | 1.40 |
| 10.53 | 3.55 | 3.29 | 3.17 | 3.64 | 2.91 | 3.09 | 1.90 | 1.40 |
| 10.88 | 3.34 | 3.26 | 3.16 | 3.64 | 2.83 | 2.93 | 1.90 | 1.40 |
| 11.23 | 3.34 | 3.24 | 3.16 | 3.64 | 2.78 | 2.83 | 1.90 | 1.40 |
| 11.69 | 3.28 | 3.23 | 3.15 | 3.64 | 2.75 | 2.75 | 1.90 | 1.40 |

| **Table S3.** *T*_1,obs_ (400 MHz) of H_2_O in D_2_O solutions at different concentrations of Gd(MT14DCH). | |
| --- | --- |
| ***T*_1,obs_ (s)** | **[Gd(MT14DCH)] (mm)** |
| 13.922 | 0 |
| 4.063 | 0.0393 |
| 2.329 | 0.0768 |
| 1.209 | 0.1545 |
| 0.608 | 0.3112 |
| 0.419 | 0.4657 |
| 0.317 | 0.6166 |

| **Table S4.** *T*_1,obs_ (80 MHz) of H_2_O in aqueous Gd(MT14DCH) solutions at different concentrations. | |
| --- | --- |
| ***T*_1,obs_ (s)** | **[Gd(MT14DCH)] (mm)** |
| 3.01 | 0 |
| 2.13 | 0.0349 |
| 1.69 | 0.0699 |
| 1.29 | 0.1397 |
| 0.45 | 0.4192 |
| 0.35 | 0.5590 |
